# Methicillin-resistant *Staphylococcus aureus* and glycopeptide-resistant enterococci in fecal samples of birds from South-Eastern Poland

**DOI:** 10.1186/s12917-019-2221-1

**Published:** 2019-12-30

**Authors:** Jolanta Kutkowska, Anna Turska-Szewczuk, Marek Kucharczyk, Halina Kucharczyk, Joanna Zalewska, Teresa Urbanik-Sypniewska

**Affiliations:** 1 Department of Genetics and Microbiology, Maria Curie-Skłodowska University, Akademicka 19, 20-033 Lublin, Poland; 2 Department of Nature Protection, Maria Curie-Skłodowska University, Akademicka 19, 20-033 Lublin, Poland; 3 Department of Zoology, Maria Curie-Skłodowska University, Akademicka 19, 20-033 Lublin, Poland

**Keywords:** *Enterococcus*, *Staphylococcus*, *Campylobacter*, *mecA*, *vanA*, *vanB*, Rook, Wild -living bird

## Abstract

**Background:**

The incidence of human infection and colonization with methicillin-resistant *Staphylococcus aureus* (MRSA) and vancomycin-resistant enterococci (VRE) has increased in the recent years. Environmental sources, including bird droppings, might play an important role as resistance reservoirs.

**Results:**

Fresh fecal samples were collected from rooks and wild-living birds during the autumn-winter period of 2016/2017, and tested for the presence of bacteria associated with human diseases. Besides bacteria representing the genera *Enterococcus*, *Campylobacter*, *Escherichia*, and *Staphylococcus*, *Enterobacter*, *Citrobacter*, *Proteus*, *Hafnia*, and *Pseudomonas* were also identified. The susceptibility of *S. aureus* and *Enterococcus* spp. isolates to methicillin, and vancomycin and teicoplanin, respectively, was analyzed to assess the avian wildlife as a reservoir of MRSA and VRE strains. Twenty-two percent of all *S. aureus* isolates were methicillin-resistant. These strains were screened by polymerase chain reaction (PCR), using the most widely used primer sets specific for the *mecA* gene. Twenty percent of all *Enterococcus* strains were phenotypically vancomycin-resistant. The presence of *van* resistance genes in these strains was investigated by PCR using *vanA* and *vanB* gene-specific primers. A good correlation between *mecA* gene detection and disc diffusion data was observed, while some discrepancy was noted between the PCR data and the vancomycin/teicoplanin phenotypic resistance pattern. The incidence of strains resistant to methicillin and glycopeptide antibiotics in wild-living birds was twice that in rooks.

**Conclusions:**

The study suggests that rooks from urban areas and passerine birds from the natural habitat carry antibiotic-resistant *Enterococcus* spp. and *S. aureus* strains, probably reflecting the presence of such isolates in the environmental food sources.

## Background

Wild-living birds are reservoirs of potentially pathogenic bacteria. They may play an important role in the epidemiology of human diseases, either directly or indirectly, by contaminating foodstuff, water, dust, or other environmental sources [1]. The risk to public health is most widely reported based on analyses of large numbers of feral pigeons whose populations greatly increased in the last few decades in European cities [2].

Free-living bird species that are frequently found in the human environment are naturally infected with viruses, bacteria, fungi, and protozoa that are also pathogenic to human [3–5]. Wild-bird populations attracted to urbanized areas can be colonized with strains of *Enterococcus* spp. [6], *Escherichia coli*, *Salmonella* spp. [7–9], and *Staphylococcus* that are all resistant to antibiotics [4, 5, 10]. *Enterococcus*, *Campylobacter*, *E. coli*, and coliforms are the predominating bacteria in feces of wild-living birds [2]. Although antimicrobial drug resistance is quite common in poultry, it has been also observed in bacteria isolated from wild birds [4].

Corvids are considered to be important environmental reservoirs and vectors of antibiotic-resistant bacterial strains [7, 8]. The rook (*Corvus frugilegus*) is an omnivore, with a Palearctic distribution. This bird leaves the roosting place during the day to search for food, usually within 0.5–2 km from the breeding colony, or even 10–40 km from the winter roost [6, 11, 12]. Currently, the rook is one of the most numerous species found in agroecosystems. Recently published evaluations of the current rook population in Poland vary, ranging from 150,000 to 200,000 pairs or from 250,000 to 310,000 [13]. Rook, on account of its increasing abundance and mobility, also plays a role in the dissemination of pathogenic microorganisms [8].

*Enterococcus*, considered a typical commensal of the intestinal tract, is fast emerging as a pathogen that causes a variety of infections, including urinary tract infections, bacteremia, endocarditis, and meningitis. It spreads to patients in the hospital or is transmitted through the food supply. Enterococci are intrinsically resistant or tolerant to many antibiotics, and can acquire drug resistance either via integrons, plasmid transfer, or transposon acquisition [14–16].

The first-line treatment of choice for systemic enterococcal infections are glycopeptide antibiotics, mainly vancomycin (Van) and teicoplanin (Tei). *Enterococcus faecalis* and *E. faecium* are most frequently described as etiological agents of nosocomial infections caused by vancomycin-resistant enterococci (VRE) [14]. Until now, nine distinct vancomycin resistance gene clusters have been described in enterococci (*vanA*, *vanB*, *vanC*, *vanD*, *vanE*, *vanG*, *vanL*, *vanM*, and *vanN*) [17]. The most predominant phenotype is VanA, characterized by high-level resistance to both vancomycin and teicoplanin. VanB-type phenotype is commonly observed among clinical isolates and in fecal strains of domestic animals. VanB strains are resistant to various concentrations of vancomycin but are susceptible to teicoplanin [14, 18].

VRE intrinsically resistant to low levels of vancomycin and containing the *vanC* gene, such as *Enterococcus gallinarum*, *E. casseliflavus*, and *E. flavescens*, have been isolated from wild bird feces [17] and from a poultry farm [19]. The presence of VRE and/or high-level aminoglycoside-resistant enterococci in wild small mammals, rabbits, and birds has been documented [6, 10, 20]. Further, the same variants of the *vanA* gene cluster associated with Tn*1546* [21] or located on a transferable broad host-range plasmid [15] have been detected in enterococci of both human and animal origin. This may indicate that resistance genes can spread among isolates from different sources [22].

The *mecA* gene is the major determinant of methicillin resistance in staphylococci, and is found in both *Staphylococcus aureus* and coagulase-negative staphylococci [23]. MRSA has been isolated from birds, including poultry, pigeons, parrots, and wild birds [4].

The aim of the current study was to evaluate and compare the presence of potentially pathogenic bacteria in the feces of rooks feeding in public sites and wild birds inhabiting places far from the urban areas, to verify whether feeding municipal food waste increases the occurrence of bacteria that could be harmful to human health. The fecal bacterial flora of rooks feeding in Lublin and the surrounding region, and Kraków (urban areas) was compared with that of wild birds that were caught and ringed in the Vistula river valley (outside urban areas). The identified potential pathogens *Enterococcus* spp. and *S. aureus* were tested for antibiotic resistance. Using antibiotic susceptibility assays, the occurrence of the *mecA* gene in *S. aureus*, and the *vanA* and *vanB* genes in enterococcal isolates from rooks and wild-living birds was determined.

## Results

### Locations of the sample collection sites and isolate culture analysis

Samples collected from rooks and wild-living migratory birds from urban areas and the Vistula valley, respectively, were analyzed using bacteriological approaches. Overall, 190 samples were obtained: 163 from rooks (five locations) and 27 samples from migratory birds representing six genera (one location) (Table 1).

**Table 1:** Overview of the sampling of rooks and other genera of wild-living birds

Species	Sampling location ^a^	Sample type	Birds (*n*)
Rook (*Corvus frugilegus*)	**A**	Feces	59
	**B**		24
	**C**		25
	**D**		25
	**E**		30
Robin (*Erithacus rubecula*)	**F**	Cloacal swab	4
Song thrush (*Turdus philomelos*)			5
Fieldfare (*Turdus pilaris*)			1
Common blackbird (*Turdus merula*)			12
Spotted flycatcher (*Muscicapa striata*)			1
Common chiffchaff (*Phylloscopus collybita*)			1
Dunnock (*Prunella modularis*)			2
Thrush nightingale (*Luscinia luscinia*)			1

^a^ **A**, Lublin 1 (51°15′00″N 22°34′00″E), a rookery and wintering site in a park in the vicinity of a hospital (A1–A59), and a rookery in a city park (L1–L12); **B**, Niemce near Lublin (51°21′40″N 22°38′16″E), a rookery in the vicinity of a communal waste dump (N1–N24); **C**, Garbów (51.35028°N 22,34,000°E), a rookery in a village park (G1–G25), **D**; Suchowola (50°35′26.8″N 23°14′52.0″E), a rookery in a village park (S1–S25); **E**, Kraków (50°03′41″N 19°56′18″E), a rookery in the Jordan’s Garden (K1–K30); **F**, Kaliszany (51°04′12.14″N 21°48′42. 01″E), the Vistula river valley (C1–C27)

The results of microbiological examination and isolate identification in fecal samples from the specified areas are summarized in Table 2. The commonly identified genera *Enterococcus* spp., *Campylobacter* spp., *E. coli*, and *S. aureus* accounted for 55% (90/163), 57% (93/163), 56.4% (92/163), and 28.2% (46/163), respectively, of all isolates from rook feces; and 81.5% (22/27), 37% (10/27), 85% (23/27), and 29.6% (8/27), respectively, of all isolates from cloacal swabs of wild birds. The overall frequency of these genera suggested that they are normal constituents of the intestinal flora of the tested bird species. Except for the similar frequency of isolation of *S. aureus* from rooks and wild-living birds (28.2% vs. 29.6%, respectively), the differences in the relative abundance of dominant genera indicated host-dependence of the intestinal microflora composition and the effect of the bird feeding habits. Four species of *Enterococcus* (*E. faecalis*, *E. faecium*, *E. casseliflavus*, and *E. raffinosus*) were distinguished by biochemical tests (EN—COCCUStest).

**Table 2:** Bacteria isolated from the feces of rooks and cloacal swabs of wild-living birds

	Rooks						Wild-living birds
	A	B	C	D	E	Total	F
*S. aureus*	15 (25.4%)	9 (37.5%)	4 (1.6%)	6 (2.45)	12 (40%)	46 (28.2%)	8 (29.6%)
*S. epidermidis*	4 (6.7%)	3 (1.3%)	–	4 (6.7%)	–	11 (6.7%)	–
*Enterococcus*	38 (64%)	14 (58%)	10 (40%)	15 (60%)	13 (43%)	90 (55%)	22 (81.5%)
*E. coli*	40 (67.7%)	16 (66.6%)	10 (40%)	11 (44%)	15 (50%)	92 (56.4%)	23 (85%)
Other *Enterobacterales* genera	23 (38.9%)	9 (37.5%)	3 (12%)	6 (96.3%)	18 (58.9%)	59 (36%)	11 (40.7%)
*Pseudomonas*	8 (13.5%)	2 (8.3%)	2 (8%)	1 (40%)	3 (10%)	16 (9.8%)	1 (3.7%)
*Campylobacter*	39 (66%)	18 (75%)	12 (48%)	10 (40%)	14 (46.6)	93 (57%)	10 (37%)
Bacterial samples (*n*)	**59**	**24**	**25**	**25**	**30**	**163**	**27**

The percentage of isolates from a specific genus among all isolates is shown in parentheses; −, not detected

*Enterobacterales* genera*: Enterobacter* spp*.*, *Cronobacter* spp*.*, *Citrobacter* spp*.*, *Kluyvera* spp*.*, *Hafnia* spp*.*, and *Proteus* spp*.*

### Antimicrobial susceptibility testing and detection of methicillin and vancomycin resistance genes

Methicillin disc diffusion and oxacillin agar tests revealed that 19.5% (9/46) of *S. aureus* isolates from rook samples and 37.5% (3/8) from wild-living birds were resistant to methicillin. The incidence of MRSA isolation from rook samples was mainly related to locations B and D [8.3% (2/24) and 8% (2/25), respectively] (Tables 2 and 3). MRSA strains were isolated from the spotted flycatcher, robin, and common blackbird.

**Table 3:** Identification of methicillin-resistance genes in MRSA isolates

Presence of a specific PCR product	Isolates (*n*)	
	Rooks	Wild-living birds
MecA1/2	3	1
MecA3/4	2	0
MecA1/2 and MecA3/4	4	2

*Enterococcus* strains resistant to vancomycin and teicoplanin represented *E. faecalis* (10 isolates) and *E. faecium* (13 isolates). Isolates of the other two species, *E. casseliflavus* and *E. raffinosus*, were sensitive to both glycopeptides. The minimal inhibitory values (MIC) of Van and Tei were determined (Table 4). The analysis confirmed the results of the disc diffusion test. According to the Clinical Laboratory Standards Institute (CLSI) criteria, six strains isolated from rooks and six strains isolated from wild birds exhibited the VanA phenotype (MIC ranges: Van ≥256 μg/ml and Tei 16–256 μg/ml). Of 23 VRE strains tested, five strains from rooks and one from a wild bird displayed the VanB phenotype (MIC ranges: Van > 256 μg/ml and Tei 1–4 μg/ml). Four strains exhibited intermediate susceptibility to vancomycin (MIC range 8–16 μg/ml) and sensitivity to teicoplanin (MIC 1–8 μg/ml); three of these strains were isolated from rooks (Table 4). Overall, combined vancomycin and teicoplanin resistance was more frequent among enterococcal isolates from wild-living birds than those from rooks. The VanA phenotype was predominant (2:1) in rooks feeding in the vicinity of a communal waste dump (location B) compared with those feeding in the municipal parks (locations A and C–E), (Table 1).

**Table 4:** Vancomycin and teicoplanin susceptibility of *Enterococcus* spp.

*Enterococcus* spp. genotype	Van/ Tei susceptibility	MIC range (μg/ml)		Isolates (*n*)	
		Van	Tei	Rooks	Wild-living birds
*E. faecalis* (*n* = 10)					
*vanA*	VanR/TeiI	256	16	1	0
*vanB*	VanR/TeiS	> 256	1–4	5	1
	VanI/TeiS	16	1	1	0
*vanA* and *vanB*	VanR/TeiR	> 256	32–64	0	2
*E. faecium* (*n* = 13)					
*vanA*	VanR/TeiI	256	16	1	0
	VanR/TeiR	> 256	128–256	3	3
*vanB*	VanR/TeiI	256	16	1	0
*vanA* and *vanB*	VanR/TeiR	> 256	32–64	1	1
no *vanA* or *vanB*	VanI/TeiS	8–16	1–8	2	1

Abbreviations: *R* resistant; *I* intermediate; *S* susceptible; *Van* vancomycin; *Tei* teicoplanin

The primers used for polymerase chain reaction (PCR)-based detection of specific antibiotic resistance genes in *S. aureus* and *Enterococcus* spp. isolates resistant to methicillin or glycopeptides, respectively, are listed in Table 5. Using two primer pairs (MecA1/2 and MecA3/4), the presence of *mecA* gene was confirmed in all MRSA rook isolates (9) and wild-living bird isolates (3). Either 310-bp or 527-bp amplicons, or both, were detected by PCR (Table 3). Among the 12 MRSA isolates, both amplicons were detected in six isolates; only the small amplicon was detected in four isolates; and only the large amplicon was detected in two isolates. In general, there was a good agreement between the PCR-based *mecA* gene detection and the results of the disc agar diffusion test. Since the approach detected both *mecA* gene products in half of the isolates screened, the PCR analysis should be performed using at least two primer sets for MRSA confirmation.

**Table 5:** Primer sequences, references, and expected amplicon size of target gene analysed in the current study

Primer	Primer sequence (5′–3′)	Amplicon size (bp)	Reference
MecA1	GTAGAAATGACTGAACGTCCGATAA	310	[24]
MecA2	CCAATTCCACATTGTTTCGGTCTAA		
MecA3	GGGATCATAGCGTCATTATTC	527	[25]
MecA4	AACGATTGTGACACGATAGCC		
vanA(+)	GGGAAAACGACAATTGC	732	[18]
vanA(−)	GTACAATGCGGCCGTTA		
vanB(+)	ACGGAATGGGAAGCCGA	647	[18]
vanB(−)	TGCACCCGATTTCGTTC		

The results of PCR used to screen glycopeptide-resistant isolates are shown in Table 4. Eight isolates tested positive for the *vanA* gene only (732-bp product); eight isolates tested positive for the *vanB* gene only (647-bp product); and four isolates displaying the VanA susceptibility pattern tested positive in both reactions. The *vanA* genotype was more prevalent in samples from wild-living birds. PCR products indicative of the presence of the *vanA* or *vanB* genes were detected in three different species of wild-living birds: the spotted flycatcher, common chiffchaff, and thrush nightingale. In three strains (*E. faecium*) exhibiting the intermediate level of resistance to vancomycin (MIC 8–16 μg/ml) and susceptibility to teicoplanin (MIC 1–8 μg/ml), neither *vanA* nor *vanB* gene products were detected.

## Discussion

In the current study, we reported the isolation and characterization of bacteria from the feces of urban rooks and cloacal swabs of wild-living bird. From both rooks and wild-living birds, methicillin- and glycopeptide-resistant strains of *S. aureus* and *Enterococcus* spp., respectively, were isolated.

In Poland, in the years 2000–2014, a moderate decline in the rook breeding population was observed; however, in the eastern part of country, the rook population was regarded as stable or increasing [13]. The rooks frequently roam in the vicinity of inhabited areas, recreation areas, and parks, thus coming in close contact with human and animals. In fact, environmental (air and water) pollution by rook feces was reported [2, 26, 27]. Infection caused by feces of wild birds can be contracted in a number of ways: by direct contact or inhalation, or by food and water contamination. Although the risk of contracting an infection from bird droppings is relatively low, diseases caused by antibiotic-resistant staphylococci and enterococci may be severe. In the current study, four bacterial genera (*Enterococcus* spp., *Campylobacter* spp., *E. coli*, and *S. aureus*) were commonly identified in rook feces collected at five different localities and in samples from wild-living birds from one region.

The occurrence of bacteria and fungi in urban rook populations in Zagreb (Croatia) during the breeding period in 2010 was described by Vlahović et al. [26]. Microbiological examination of fresh fecal samples revealed the presence of *E. coli*, *Bacillus* spp., *Staphylococcus* spp., *Streptococcus* spp., *Agrobacterium radiobacter*, and *Acinetobacter* spp. [3, 26, 28]. In the current study, no *Salmonella* isolates were detected, although *S.* Enteritidis was found in the feces of rooks wintering in Czechia in another study [7]. Further, one study of *Salmonella* prevalence in free-living birds in southern Poland reported no *Salmonella* isolation from the feces or tissue of *Corvus cornix*; by contrast, 16.6% of samples from *C. frugilegus* were positive [29].

The high frequency of detection of *Campylobacter* species (57% of all rook isolates) noted herein was not described in other reports. For example, in a study conducted in Zagreb, only one rook sample out of 57 fecal samples was positive for *C. jejuni* [26]. Nonetheless, other studies suggest that *C. jejuni* may be a typical component of the intestinal flora of at least some bird species [4]. *Campylobacter* spp. that could potentially be transmitted to human have been isolated from migrating ducks, pigeons, passerine birds, and crows (*Corvus*) [1]. Phylogenetic analyses of free-ranging American crows confirmed the notion that some *C. jejuni* strains potentially associated with disease might be shared by crows, human, poultry, and livestock [30].

Rooks could be important vectors for the dissemination of antibiotic-resistant enterococci [6] and MRSA [28] in Europe because they are scavengers and cover relatively long distances. Corvids and gulls that feed on garbage in urbanized areas are also considered to be environmental reservoirs and vectors of resistant isolates [8]. In the current study, we noted a relatively high frequency of isolation of *Enterococcus* spp. in rook samples (55%) and in wild-living birds (81.5%) in the examined localities in south-east Poland (Table 2).

The prevalence of *S. aureus* in fecal samples from rooks and wild birds was comparable (Table 2), but the average incidence of methicillin-resistant isolates was two times higher among isolates from wild-living birds than those from rooks (Table 3). Loncaric et al. [28] observed significant difference in the occurrence of MRSA isolates between migratory population compared with resident population of *C. frugilegus* from eastern Europe.

In the current study, glycopeptide-resistant strains were detected relatively more frequently in samples from rook feces collected in the vicinity of communal waste dumps (location B) than in city (locations A and E) or village (locations C and D) parks. Samples from wild-living birds contained two times more glycopeptide-resistant enterococci than fecal samples from rooks (36.4% vs. 16.6%, respectively). Since it was not possible to obtain the feces of wild bird species, the only available material was cloacal swabs. It is possible that the sampling site may have slightly affected the final results. The prevalence of VRE in rooks in individual countries may differ significantly, with Poland (14% in Gdynia and 8% in Jaroslaw) and Czechia (10%) as countries with the most frequent VRE isolation [6].

The results of the disc agar diffusion test and PCR determination of the presence of resistance genes were in good agreement. Specifically, the presence of the *mecA* gene was confirmed in all phenotypically resistant *S. aureus* isolates, whereas the *vanA* and/or *vanB* genes were identified in 83% of phenotypically vancomycin- and teicoplanin-resistant, or intermediate-resistant *Enterococcus* spp. isolates. In the PCR analysis, most strains yielded only one amplicon, which was consistent with the phenotypic resistance pattern. The amplicons from both, *vanA* and *vanB* genes, in strains with the VanA resistance phenotype were mainly detected in enterococci from wild-living birds. The amplicons from these two genes were also detected in VanA phenotypically resistant clinical isolates of *E. faecium* and *E. faecalis* [31], and in VanB phenotype *E. faecium* [32].

Examination of VRE presence in fecal samples of rooks wintering in Europe resulted in the identification of 6% of 1073 enterococci growing on a selective medium as *E. faecium*, of which 12.5% harbored the *vanA* and *ermB* (macrolide resistance) genes [6]. Enterococci with plasmid-encoded glycopeptide-resistance genes *vanA*, *vanB*, and *vanM* are reservoirs for the transmission of these genes by conjugation to other enterococci and to other Gram-positive cocci, including *S. aureus* [6, 33, 34].

Based on the findings of the current study, bacterial colonization of the host bird is associated with its feeding and breeding place. The obtained data may be used to map the dissemination of antibiotic-resistant strains by urban rooks and other wild birds, and to track the possible consequences of transferring the resistance determinants to other animals or to human.

## Conclusions

Bacteria from the genera *Campylobacter*, *Enterococcus*, *Escherichia*, and *Staphylococcu*s are predominant in the intestinal flora of rooks and in eight species of other wild-living migratory birds. Other genera, e.g., *Enterobacter*, *Citrobacter*, *Hafnia*, *Cronobacter*, *Kluyvera*, *Proteus*, *Morganella*, and *Pseudomonas*, are identified much less frequently. Among *S. aureus* isolates, 19.6% from rooks and 37.5% from wild-living birds were resistant to methicillin. *Enterococcus* isolates carrying *van* resistance genes were identified with a frequency of 14.4% (13/90) and 31.8% (7/22) in samples from rooks and wild birds, respectively. The overall frequency of isolation of strains resistant to methicillin and glycopeptide antibiotics in wild-living bird samples was approximately two times higher than that in rook samples.

## Methods

### Bird sampling

To obtain rook samples, swabs were taken from the top surface of fresh feces. Cloacal swabs were collected from eight different species of wild-living migratory birds of the order *Passeriformes* at the time of ringing, between 1 September 2016 and 31 October 2017.

The ornithologist ringed the birds with the consent of the General Directorate of Environmental Protection, Poland (nos. 291/2016 and 291/2017) in accordance with decisions DZP-WG.6401.03.98.2016.km, DZP-WG.6401.03.97.2017.jro, and Minister of Environment DLP-VIII-6713-21/29762/14/RN. In accordance with the Polish law, the Act of January 15th 2015 on the Protection of Animals Used for Scientific or Educational Purposes (Dz.U.2015.266), fecal and cloacal sampling does not require the consent of the local ethics committee (Article 1.2, point 5).

The samples (*n* = 190) were collected at six localities in south-east Poland (Table 1). Samples were collected using Amies clear gel collection and transport swabs (BioMerieux, Marcy-l’Etoile, France), and stored on ice (4–7 °C) for 1–7 h prior to culture. Individual samples were placed overnight in buffered peptone water (Biomaxima, Gdańsk, Poland) at 35–37 °C, and then inoculated onto selective media.

Hektoen enteric agar, MacConkey agar, and triple sugar iron agar (BioMerieux) were used for the recovery of Gram-negative gastrointestinal pathogens (*Salmonella* and *Shigella*) and other intestinal genera (*E. coli*, *Klebsiella*, and *Enterobacter*). All isolates were processed using API 20E strips (BioMerieux), following the manufacturer’s instructions. Supplementary investigation of cytochrome oxidase was performed using Oxidase Test (Merck, Darmstadt, Germany). β-glucuronidase test with the substrate 4-methylumbelliferyl-beta-d-glucuronide (BD Difco), Rapid Enterococci ChromoSelect agar (Sigma, St Louis, MO), *Enterococcus*-selective agar (Slanetz-Bartley) (BTL, Łódź, Poland), bile-esculin agar (Merck), and EN-COCCUStest (Pliva-Lachema, Brno, Czechia) based on acid production from sorbitol, l-arabinose, mannitol, sorbitol, mellibiose, raffinose, melezitose, and production of arginine dihydrolase, to isolate and identify enterococci. Further, mannitol salt agar (BTL) was used for the isolation of pathogenic staphylococci. *S. aureus* isolates were identified using colony morphology, catalase test, API Staph biochemical tests (BioMerieux), the ability to coagulate rabbit plasma, and based on clumping factor production. Campylosel agar (BioMerieux), a selective medium for the isolation of intestinal *Campylobacter* (mainly *C. jejuni* and *C. coli*) from stools, was used. *Campylobacter* isolation was performed following standard conditions, after a 24-h enrichment period at 42 °C under micro-aerobic conditions in a CampyPak Plus microaerophilic system with a palladium catalyst (BD Diagnostic Systems, Sparks, MD).

### Antibiotic susceptibility testing

Susceptibility to antibiotics was tested on Mueller Hinton agar (Biomaxima) by using the Kirby-Bauer disc diffusion method, and commercially prepared discs of 30 μg vancomycin, 30 μg teicoplanin, and 5 μg methicillin (Liofilchem, Italy), according to CLSI guidelines [35]. Plates were incubated at 35 °C for 24 h. *S. aureus* strains identified as methicillin resistant by disc diffusion assay were then tested by oxacillin agar screening. Specifically, a colony suspension was spotted on MH agar supplemented with 4% NaCl and oxacillin at 6 μg/ml (Sigma), according to CLSI recommendations. After incubation for 24 h, any growth was interpreted as a positive result for oxacillin resistance [36]. MIC values for vancomycin and teicoplanin (Sigma) were tested by broth microdilution method, according to CLSI guidelines [37]. The final concentrations of vancomycin and teicoplanin ranged from 0.125 to 256 μg/ml.

### Primers

The *mecA* gene was PCR-amplified using primers MecA1 and MecA2 [24], and MecA3 and MecA4 [25]. The *vanA* gene was PCR-amplified using primers VanA(+) and VanA(−). The *vanB* gene was PCR-amplified using primers VanB(+) and VanB(−) [18] (Table 5).

### PCR

The presence of the *mecA* gene, and *vanA* and *vanB* genes was determined by PCR in MRSA and *Enterococcus* spp. isolates exhibiting phenotypical vancomycin resistance, respectively. The template DNA was isolated and purified using the Wizard genomic DNA purification kit (Promega, Madison, WI) supplemented with either lysostaphin (5 mg/ml; Sigma), for MRSA, or lysozyme (10 mg/ml, Sigma), for enterococci, as described elsewhere [18]. PCR was performed in a 25-μl reaction mixture containing template DNA (10–200 ng), 200 μg of each dNTP, 1.5 mM MgCl_2_, 20 mM Tris-HCl (pH 8.4), 50 mM KCl, 200 nM (each) primer, and 2.5 U of *Taq* polymerase (Sigma). Thermal cycling for *vanA* detection was conducted using the following program: an initial denaturation step at 96 °C for 180 s; followed by 35 cycles of 94 °C for 45 s, 46 °C for 45 s, and 72 °C for 60 s; and a final extension step at 72 °C for 360 s. The *vanB* and *mecA* genes were amplified using the same protocol, except that the annealing temperature used was 50 °C and 54 °C, respectively. PCR products were visualized on 1% agarose gel.

The following strains were used as positive and negative controls: *S. aureus* ATCC 43300, methicillin R, *mecA* gene positive control; *S. aureus* ATCC 25923, methicillin and vancomycin S, negative control; *E. faecium* ATCC 51559, vancomycin and teicoplanin R, *vanA* gene positive control; *E. faecalis* ATCC 51299, vancomycin R and teicoplanin S, *vanB* gene positive control; *E. faecalis* ATCC 29212, vancomycin S, negative control.

## Data Availability

The datasets generated during and/or analyzed during the current study are available from the corresponding author on reasonable request.

## Abbreviations

CLSI: Clinical and Laboratory Standards Institute
MH: Mueller Hinton
MIC: Minimal inhibitory concentration
MRSA: Methicillin-resistant *Staphylococcus aureus*
PCR: Polymerase chain reaction
Tei: Teicoplanin
Van: Vancomycin
VRE: Vancomycin-resistant enterococcus
